# Clinical characteristics and outcomes of patients with mental illnesses who attempted suicide by drug overdose: A retrospective analysis of 109 cases

**DOI:** 10.1002/brb3.3058

**Published:** 2023-05-24

**Authors:** Naiyun Chen, Shaoli Li, Shu Huang, Jianbo Lai, Duo Lv

**Affiliations:** ^1^ Department of Emergency Medicine the First Affiliated Hospital, Zhejiang University School of Medicine Hangzhou China; ^2^ Department of Medical Oncology, the Second Affiliated Hospital Zhejiang University School of Medicine Hangzhou China; ^3^ Department of Psychiatry, the First Affiliated Hospital Zhejiang University School of Medicine Hangzhou China; ^4^ The Key Laboratory of Mental Disorder's Management in Zhejiang Province Hangzhou China; ^5^ Department of Clinical Pharmacy, the First Affiliated Hospital Zhejiang University School of Medicine Hangzhou China

**Keywords:** drug overdose, emergency care, mental illness, suicide

## Abstract

**Objective:**

Individuals with mental illnesses are exposed to an increased risk of suicide. In this study, we aimed to investigate the clinical characteristics and outcome of psychiatric patients who attempted suicide by drug overdose and required emergency care.

**Methods:**

A retrospective study was carried out in the Department of Emergency, the First Affiliated Hospital, Zhejiang University School of Medicine. Electronic medical records of psychiatric patients who were hospitalized due to suicide attempts from March 2019 to February 2022, with a discharge diagnosis of drug overdose were reviewed. Suicide‐related data of patients were collected, including suicide month, time from suicide to admission, type of drugs, the number of tablets taken, as well as demographic and clinical profiles (e.g., gender, age, marital status, profession, physical comorbidities, and diagnosis of mental illness).

**Results:**

In the results, half of the patients were young people, female patients accounted for a higher proportion (72.5%), and the incidence of suicide was higher in winter than other seasons. Among the 109 psychiatric patients, 60 patients (55.0%) had a history of major depressive disorder, and 86 patients (78.9%) committed suicide with various psychotropic drugs, among which anxiolytics were the most commonly used drugs. Thirty‐seven patients (33.9%) experienced severe physical complications caused by drug overdose, with lung infections being the most common. The clinical outcome of most patients was favorable following emergent treatment, while 2 patients (1.8%) older than 80 failed to survive.

**Conclusion:**

A better understanding of psychiatric patients referred to emergency care due to suicide by drug overdose helps to improve the clinical management and prognosis of patients.

## Graphical abstract

In this study, we characterized the demographic and clinical profiles of psychiatric patients who committed suicide by drug overdose. Although the clinical outcome of these patients is generally favorable, an emergency care is still of great importance. In this study, we also propose a clinically useful management flowchart in dealing with these patients in the emergency setting.

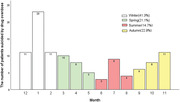

## 1 INTRODUCTION

Suicide is a major public health issue worldwide. According to data released by World Health Organization (WHO), about 800,000 to 1 million deaths per year are due to suicide, and it was noted as the second leading cause of death in the young age group (15∼29 years old) globally (WHO Mental Health). Notably, mental illnesses are important risk factors for suicidal behavior (Dahale et al., 2017; Qin, 2011), and a wide range of mental illnesses increase the risk of experiencing suicide ideation (Callanan & Davis, 2012). A recent systematic review and meta‐analysis suggested that individuals with mental illness had a nearly eight‐fold increased risk of suicide compared with those without mental illness (Too et al., 2019). More precisely, patients with major depressive disorder, bipolar disorder, or schizophrenia, respectively, had an estimated 20‐fold, 17‐fold, or 13‐fold greater risk of suicide compared with that of the general population (Chesney et al., 2014).

There are several researches studying the characteristics of suicide in patients with different mental illnesses (Lang et al., 2016; Qin, 2011; Zeppegno et al., 2015). Miller et al. found that the suicide rate of patients with bipolar disorder may be related to various factors, such as gender, age, disease severity, and disease subtype (Miller & Black, 2020). By investigating the characteristics of individuals who had attempted suicide by drug overdose, with special attention on the amount of drugs taken, Manabu et al. showed that the psychiatric outpatient history seemed not to be a risk factor for suicide attempts by ingesting higher doses of drugs (Yasuda & Kobayashi, 2019).

Suicide attempt refers to a nonfatal, self‐directed, potentially injurious behavior with any intent to die (O'Connor et al., 2013). Patients with mental illnesses are more accessible to psychotropic drugs, which may be overdosed as the culprit for suicide. At present, there is no study that specifically characterizes the clinical profiles of patients with mental illnesses who attempt suicide by drug overdose. Herein, we hypothesized that patients with mood disorders were at a greater risk of suicide by overdosing psychotropic drugs. In the current study, we thus aimed to address the clinical characteristics and outcome of this population, and proposed a diagnosis and treatment framework for clinical management.

## 2 METHODS

This retrospective study enrolled hospitalized patients from the Department of Emergency, the First Affiliated Hospital, Zhejiang University School of Medicine from March, 2019 to February, 2022. Due to the anonymous, retrospective, and nonintervention nature of this study, informed consent from all patients was waived and was approved by the Clinical Research Ethics Committee of the First Affiliated Hospital, Zhejiang University School of Medicine (Approval number: 2021‐IIT‐513).

### 2.1 Study subjects

With the assistance of the Electronic Case Record System, patients with a discharge diagnosis of drug overdose were screened. Subjects met the following criteria were included (1) suicide attempt by drug overdose with their own medications and (2) a preexisting diagnosis of any mental illness before suicide attempt.

### 2.2 Data collection

For patients enrolled, clinical profiles such as the suicide month, time from suicide to admission, types of drugs (psychotropic drugs, e.g., antidepressants, anxiolytics, antipsychotics, anticonvulsants, mood stabilizers, and nonpsychotropics drugs) and the number of tablets taken were collected. We also recorded the demographic and other data, including gender, age, marital status, profession, physical comorbidities, and diagnosis of mental illnesses.

### 2.3 Statistical analyses

The statistical analysis was performed with SPSS 25.0 for Windows (SPSS, Inc., Chicago, IL). The categorical variables were presented as numbers (frequency). The continuous variables were presented as mean ± standard deviation (SD) or Min‐Max values.

## 3 RESULTS

### 3.1 Demographic and clinical characteristics

**FIGURE 1:**
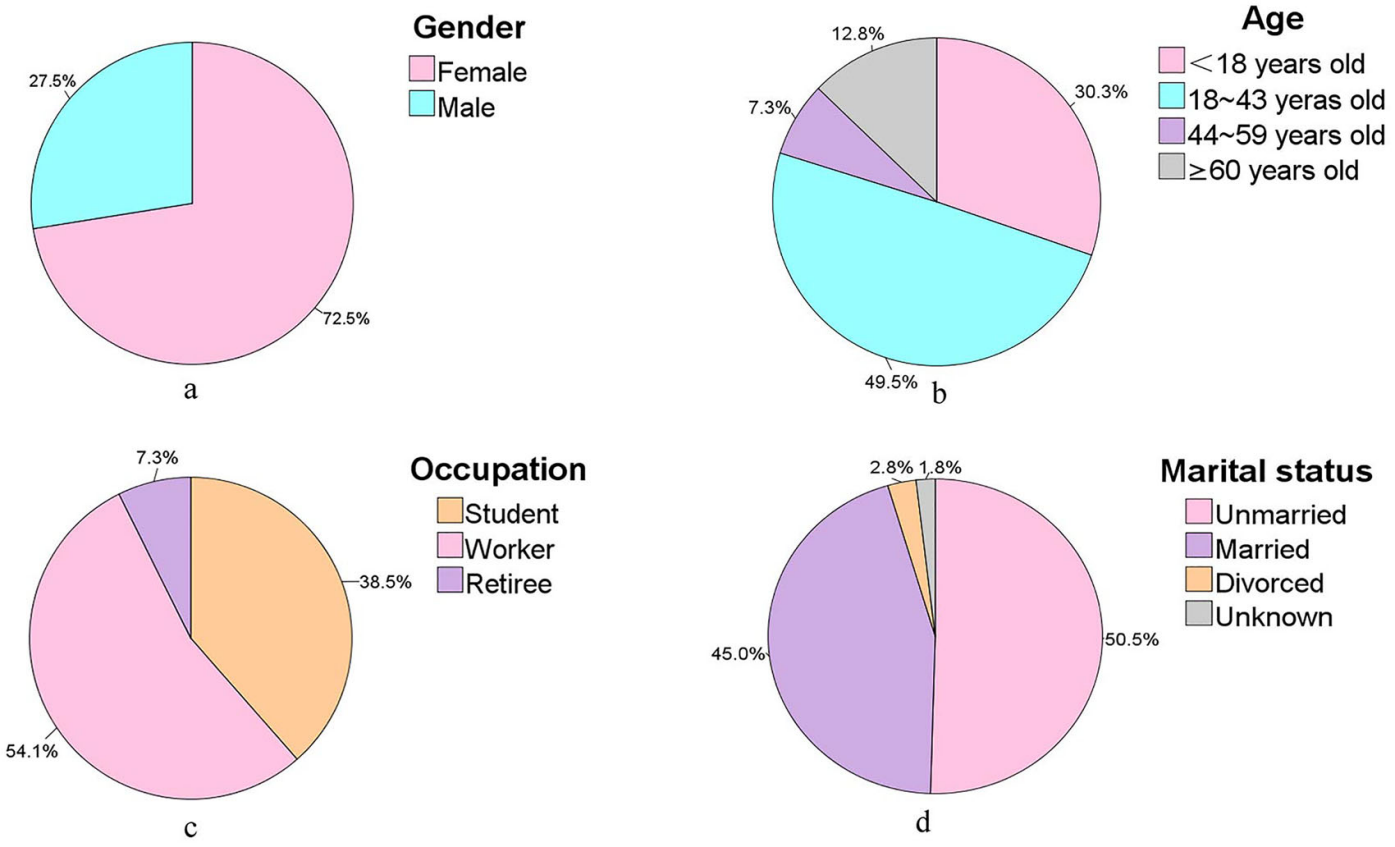
Demographic profiles of patients (*n* = 109). (a) Gender distribution of patients. (b) Age distribution of patients. (c) Occupational distribution of patients. (d) Marital status of patients.

In total, 109 cases meeting the inclusion criteria were included in the final analysis. Of these patients, 79 (72.5%) were women, 30 (27.5%) were men, and the male to female ratio was 1:2.63. The age of all patients ranged between 12 and 87 years old, with an average age of 31.3 ± 18.8 years old. According to the WHO's classification of age groups, there were 33 cases (30.3%) in the juvenile group (< 18 years old), 54 cases (49.5%) in the youth group (18‐43 years old), 8 cases (7.3%) in the middle‐aged group (44‐59 years old), and 14 cases (12.8%) in the elderly group (≥60 years old). Other demographic profiles on individual occupation and marital status were also shown in Figure 1. Twenty‐six patients had other physical illnesses, of which 13 had comorbid hypertension, with a mean age of 63.3 ± 5.2 years old.

### 3.2 History of mental illnesses

**FIGURE 2:**
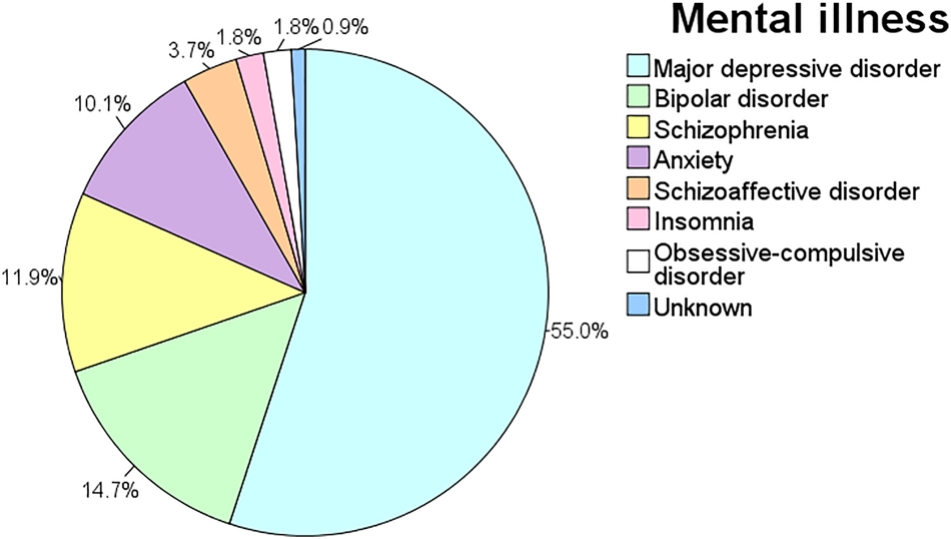
Frequency of different mental illnesses in patients (*n* = 109).

**FIGURE 3:**
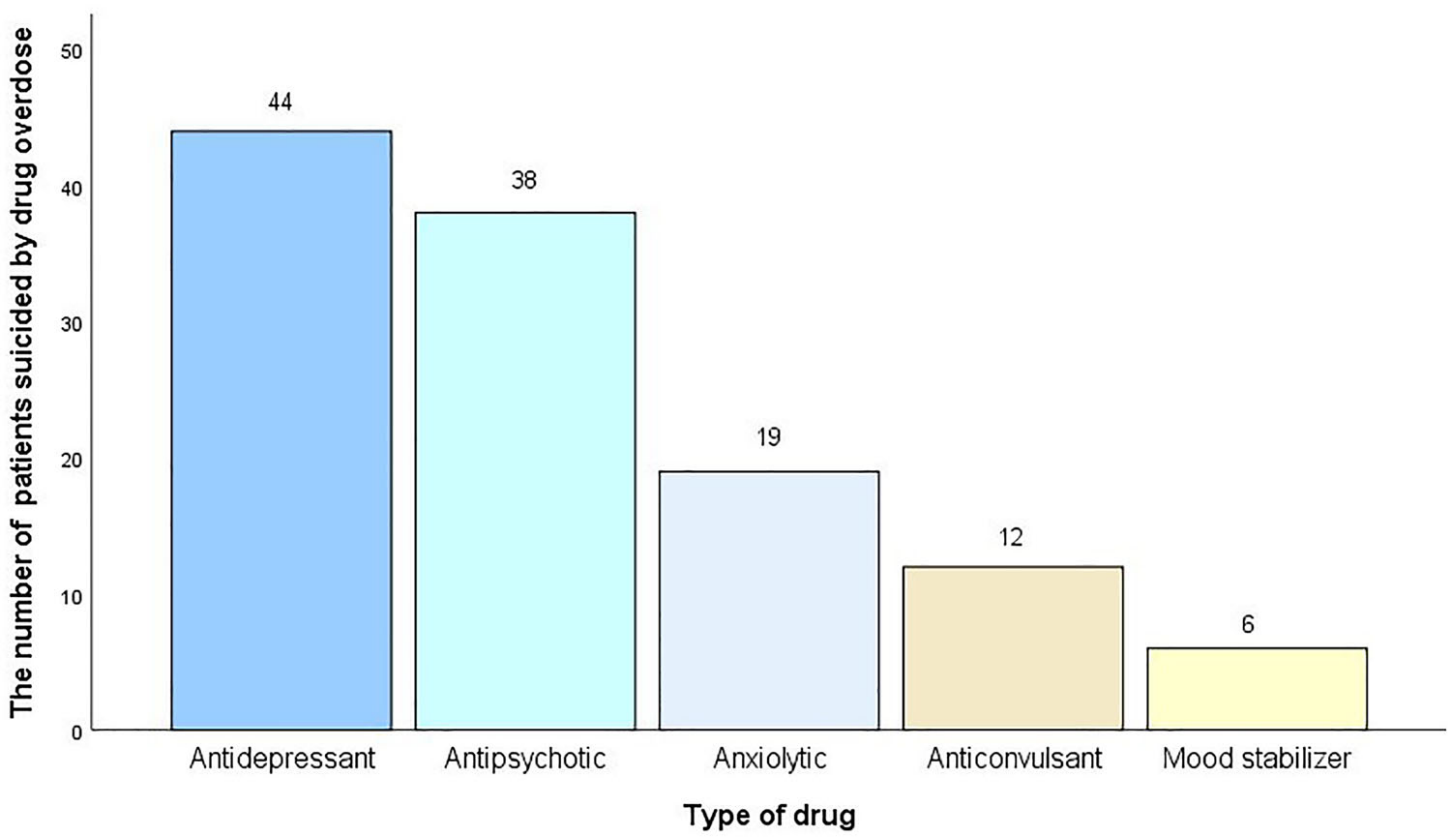
Psychotropic drugs used for treat mental illnesses in patients with a clear medication history (*n* = 72).

Among the 109 patients, 60 patients (55.0%) were previously diagnosed with major depressive disorder and 16 patients (14.7%) were diagnosed with bipolar disorder. The preexisting types of psychiatric diagnosis before suicide were shown in Figure 2. Seventy‐two patients (66.1%) reported a clear history of drug treatment for mental illnesses. Thirty patients (27.5%) were treated with monotherapy, and 42 patients (38.5%) were treated with at least two agents. Twenty‐four patients (22.0%) were untreated, and the medication histories of the remaining 13 patients (11.9%) were unknown. Among the patients treated with monotherapy, 14 patients (12.8%) were treated with an antidepressant. Among the patients treated with multidrugs, 27 patients (24.8%) were medicated with a combination of two drugs (commonly one antidepressant and one antipsychotic). A total of 14 patients (12.8%) were treated with three drugs. Antidepressants, anxiolytics, and antipsychotics were the most commonly used agents. Of the 72 patients receiving drug treatment, 44 patients (61.1%) used antidepressants, 38 patients (52.8%) used antipsychotics, and 19 patients (26.4%) used anxiolytics, as shown in Figure 3.

### 3.3 Suicide‐related variables and clinical management

#### 3.3.1 Season and month distribution of suicide by drug overdose

**FIGURE 4:**
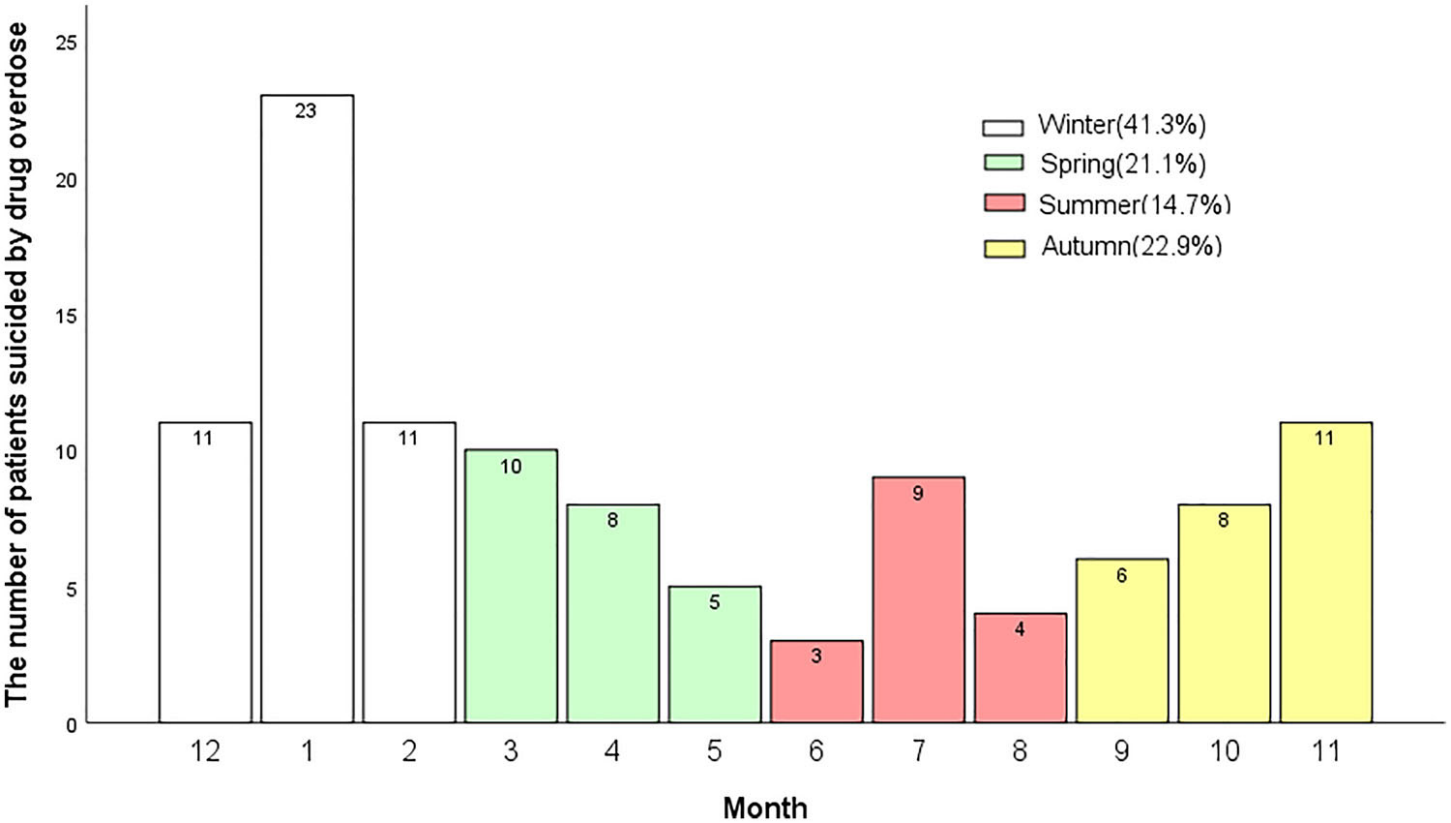
Month and season distribution of suicide by drug overdose in psychiatric patients (*n* = 109).

Twenty‐three (21.1%) patients attempted suicide in spring, 16 (14.7%) in summer, 25 (22.9%) in fall, and 45 (41.3%) in winter. Of note, most suicide attempts happened in January (see Figure 4).

#### 3.3.2 Drugs used for suicide

Eighty‐six patients (78.9%) attempted suicide with psychotropic drugs, 21 patients (19.3%) chose nonpsychotic drugs, such as colchicine and hypertension drugs, and the drugs taken by the remaining 2 patients (2.3%) were unknown. The psychiatric drugs used mainly included antidepressants, anxiolytics, antipsychotics, mood stabilizers, and anticonvulsants, among which anxiolytics were the most commonly used (47 patients) (see Table 1).

**TABLE 1:** Types and amounts of psychotropic drugs used for suicide

Drug type	Number of patients	Maximum dose/tablet	Minimum dose/tablet
Antidepressant	35	50	6
Anxiolytic	47	68	3
Antipsychotic	35	30	2.5
Mood stabilizer	4	30	6
Anticonvulsant	9	200	2

#### 3.3.3 Time for being sent to the hospital

The shortest time for a patient who attempted suicide from taking the drug to arrive at the hospital for treatment was 0.3 h, the longest was 22 h, and the average was 5.25±5.32 h.

#### 3.3.4 Complications due to drug overdose

Thirty‐seven patients suffered from severe physical complications due to drug overdose. Among them, 12 patients (32.4%) reported lung infections, 11 patients (29.7%) had electrolyte disturbance (mostly as hypokalemia), 9 patients (24.3%) had liver function impairment, 7 patients (18.9%) experienced disturbance of consciousness, 6 patients (16.2%) developed pleural effusion, 5 patients (13.5%) suffered from kidney function impairment, 2 patients (5.4%) developed toxic encephalopathy, and 2 patients (5.4%) reported cardiac arrest.

#### 3.3.5 Clinical management and outcome

After admission, 55 patients (50.5%) received gastric lavage and emetic therapy, 37 patients (33.9%) received blood perfusion. Among all patients, 67 patients (61.5%) received psychiatric consultation. Following the emergency care, the physical condition of 101 patients (92.6%) improved, 2 patients (1.8%) died in the hospital, and the clinical outcomes of 6 patients (5.5%%) were not clearly recorded. Sixty‐nine patients (63.3%) went home directly after being discharged from the hospital, 25 patients (22.9%) were transferred to the Department of Psychiatry of our hospital for further treatment, 9 patients (8.3%) were transferred to other psychiatric specialized hospitals for modified electroconvulsive therapy, and 3 patients (2.8%) were discharged automatically.

## 4 DISCUSSION

In this study, we presented the demographic and clinical characteristics of patients with preexisting mental illnesses who attempted suicide by drug overdose. Although the clinical outcomes of most patients were favorable following emergency care, early prevention and effective management of drug overdose is still challenging in this population, especially in young females with major depressive disorder and bipolar disorder.

Our research showed that females accounted for a significant higher proportion than males among all included patients. We also found that the majority of suicide attempts by drug overdose were young people (18–43 years old), which was consistent with previous studies (Yasuda & Kobayashi, 2019). A previous scoping review found that over‐the‐counter analgesics and hypnotics were frequently used by the females, the young, and people with mental disorders for self‐harm and suicidal behaviors (Shoib et al., 2022). A possible explanation is that young patients are more likely to suffer from depressive episodes and need to cope with higher life stress. In our samples, the incidence of suicide by drug overdose in winter was higher than other seasons. This was not consistent with previous researches showing that spring and summer were the peak seasons of suicide (Galvão et al., 2018; Yang et al., 2019). Zeppegno and colleagues also found a greater risk of suicide during the warmer months of the year (from April to September) (Zeppegno et al., 2015 ). The divergence of these findings may be attributed to the climate and cultural differences in different countries. Among the 109 patients, more than half of these patients had a preexisting diagnosis with major depressive disorder, and 14.7% with bipolar disorder. Therefore, our data suggests that young and female patients with a preexisting mood disorder are possibly at a higher risk of attempting suicide by drug overdose, which warrants more attention and supervision from healthcare providers and caregivers. When the patient is still in the acute phase of depressive episodes, a comprehensive and dynamic assessment of suicidal risk is necessary. In our study, nearly 80% patients did suicide by psychotropic drugs. Of special note, compared to the general population, patients with mental illnesses are more accessible to psychotropic drugs, which would be used as the culprit for drug overdose. We recommend that only a limited number of drugs would be prescribed when the mental status of the patient is still unstable. In view of early prevention, therefore, guardians or other caregivers of patients with mental illnesses are responsible to supervise the drugs. Complications occurred in about one‐third of patients, mainly involving the lung, kidney, and liver, suggesting that the adverse effects of drug overdose on these organs need full evaluation.

In our study, 2 patients failed to survive, although emergency care was immediately administered. An 87‐year‐old woman with a history of hypertension and schizophrenia was admitted to the hospital half an hour after taking 15 tablets of estazolam (1 mg per tablet) and 10 tablets of carbamazepine (200 mg per tablet). She received hemoperfusion after admission. However, she encountered cardiac arrest but did not respond to cardioversion. Another patient was an 82‐year‐old woman with a history of hypertension and cerebral infarction. She was previously diagnosed with a certain mental illness, but the specific diagnosis was unclear. She was taken to the hospital 3 h after taking 11 tablets of colchicine (1 mg per tablet). In the Department of Emergency, she suffered from severe complications including lactic acidosis, acute kidney injury, and abnormal liver function. She also received hemoperfusion after admission, but failed to survive due to cardiac arrest even with cardiopulmonary resuscitation and intravenously adrenaline treatment.

A standardized guideline may benefit the clinical management of psychiatric patients with acute drug overdose. Herein, we propose a stepwise operable working framework for clinically managing suicide due to drug overdose in patients with mental illnesses in the emergency setting. Immediate inquiry of exposure to certain drugs is the first and foremost step. For patients with unstable vital signs, first‐aid should be promptly initiated in the Emergency Department. Comprehensive laboratory and auxiliary examinations help to identify the potential adverse effects secondary to drug overdose and guide subsequent treatment choices. A psychiatric consultation is necessary and suggested. Importantly, the psychiatric diagnosis needs to be further confirmed, and for patients with predictable high suicide risk, modified electroconvulsive therapy is recommended when the physical condition remains stable. In addition to the symptomatic and supportive treatment, the implementation of detoxification treatment depends on the type of drug and the severity of the condition. The vital signs and key laboratory examinations closely linked to the culprit drug should be constantly monitored before the patient is out of danger. Although further management of the psychiatric illness is recommended when the patient recovers and is ready for discharging from the Emergency Department, a significant proportion of patients may go straight home, and the risk of suicide attempts remains high.

Several limitations of this study need to be addressed. First, this is a cross‐sectional, retrospective study, which precludes the establishment of firm causal inferences. Second, although this study aims to investigate the characteristics of psychiatric patients who attempted suicide by drug overdose, psychiatric patients without suicide attempts were not enrolled for comparison. In addition, our sample is small, and further researches with larger samples and better designs are needed to verify our findings and explore more detailed information on suicide behaviors among psychiatric patients with drug overdose.

In conclusion, we comprehensively depicted the demographic and clinical profiling of suicide by drug overdose among psychiatric patients and proposed a clinically useful flowchart for dealing with such conditions. Given the high risk of suicide in patients with mental illnesses, preventive interventions and early warning systems need to be constructed.

## AUTHOR CONTRIBUTIONS

DL and JBL conceived and designed the study. NYC and SLL conducted the literature search and wrote the first draft of the manuscript. HS conducted the data extraction. Statistical analyses were conducted by SLL under the supervision of JBL and DL. All authors contributed to and have approved the final manuscript.

## CONFLICT OF INTEREST STATEMENT

All authors claimed that there was no conflict of interest.

### PEER REVIEW

The peer review history for this article is available at https://publons.com/publon/10.1002/brb3.3058.

## Data Availability

The data that support the findings of this study are available from the corresponding author upon reasonable request.

## References

[brb33058-bib-0001] Callanan, V. J. , & Davis, M. S. (2012). Gender differences in suicide methods. Social Psychiatry and Psychiatric Epidemiology, 47(6), 857–869. 21604180 10.1007/s00127-011-0393-5

[brb33058-bib-0002] Chesney, E. , Goodwin, G. M. , & Fazel, S. (2014). Risks of all‐cause and suicide mortality in mental disorders: A meta‐review. World Psychiatry, 13(2), 153–160. 24890068 10.1002/wps.20128 PMC4102288

[brb33058-bib-0003] Dahale, A. , Sherine, L. , & Chaturvedi, S. K. (2017). In‐patient suicide in psychiatry ‐ an Indian experience. Epidemiology and Psychiatric Sciences, 26(5), 565–569. 28357967 10.1017/S2045796017000129 PMC6999019

[brb33058-bib-0004] Galvão, P. V. M. , Silva, H. R. S. E. , & Silva, C. M. F. P. D. (2018). Temporal distribution of suicide mortality: A systematic review. Journal of Affective Disorders, 228, 132–142. 29247901 10.1016/j.jad.2017.12.008

[brb33058-bib-0005] Lang, F. U. , Hubel, N. , Kösters, M. , Messer, T. , Dinse‐Lambracht, A. , & Jäger, M. (2016). Suicidality in emergency medicine: Results from a retrospective analysis of emergency documentation forms. Neuropsychiatry, 30(2), 69–73. 10.1007/s40211-016-0181-2 27287928

[brb33058-bib-0006] Miller, J. N. , & Black, D. W. (2020). Bipolar disorder and suicide: A review. Current Psychiatry Reports, 22(2), 6. 31955273 10.1007/s11920-020-1130-0

[brb33058-bib-0007] O'Connor, E. , Gaynes, B. , Burda, B. U. , Williams, C. , & Whitlock, E. P. (2013). Screening for suicide risk in primary care: A systematic evidence review for the U.S. Preventive Services Task Force [Internet]. Rockville (MD): Agency for Healthcare Research and Quality (US); Report No.: 13‐05188‐EF‐1. 23678511

[brb33058-bib-0008] Qin, P. (2011). The impact of psychiatric illness on suicide: Differences by diagnosis of disorders and by sex and age of subjects. Journal of Psychiatric Research, 45(11), 1445–1452. 21722920 10.1016/j.jpsychires.2011.06.002

[brb33058-bib-0009] Shoib, S. , Patel, V. , Khan, S. , Armiya'u, A. Y. , Saeed, F. , Swed, S. , Das, S. , & Chandradasa, M. (2022). Over‐the‐counter drug use in suicidal/self‐harm behavior: Scoping review. Health Science Reports, 5(3), e662. 35620537 10.1002/hsr2.662 PMC9128395

[brb33058-bib-0010] Too, L. S. , Spittal, M. J. , Bugeja, L. , Reifels, L. , Butterworth, P. , & Pirkis, J. (2019). The association between mental disorders and suicide: A systematic review and meta‐analysis of record linkage studies. Journal of Affective Disorders, 259, 302–313. 31450139 10.1016/j.jad.2019.08.054

[brb33058-bib-0011] WHO Mental Health . Prevention of suicidal behaviours: A task for all. Available online: http://www.who.int/mental_health/prevention/suicide/background (accessed on 27 May 2022)

[brb33058-bib-0012] Yang, C. T. , Yip, P. S. F. , Cha, E. S. , & Zhang, Y. (2019). Seasonal changes in suicide in South Korea, 1991 to 2015. PLoS One, 14(6), e0219048. 31251776 10.1371/journal.pone.0219048 PMC6599115

[brb33058-bib-0013] Yasuda, M. , & Kobayashi, T. (2019). Suicide attempts by drug overdose at Jichi Medical University Hospital emergency centre: A study of patient characteristics and quantity of drug overdose. Asian Journal of Psychiatry, 41, 34–37. 30318306 10.1016/j.ajp.2018.10.015

[brb33058-bib-0014] Zeppegno, P. , Gramaglia, C. , Castello, L. M. , Bert, F. , Gualano, M. R. , Ressico, F. , Coppola, I. , Avanzi, G. C. , Siliquini, R. , & Torre, E. (2015). Suicide attempts and emergency room psychiatric consultation. Bmc Psychiatry [Electronic Resource], 15, 13. 25652192 10.1186/s12888-015-0392-2 PMC4327969

